# A modified chain binomial model to analyse the ongoing measles epidemic in Greece, July 2017 to February 2018

**DOI:** 10.2807/1560-7917.ES.2018.23.17.18-00165

**Published:** 2018-04-26

**Authors:** Theodore Lytras, Theano Georgakopoulou, Sotirios Tsiodras

**Affiliations:** 1 Hellenic Centre for Disease Control and Prevention, Athens, Greece; 2 4th Department of Internal Medicine, Attikon University Hospital, University of Athens Medical School, Athens, Greece

**Keywords:** measles, outbreaks, modelling, surveillance, epidemiology

## Abstract

Greece is currently experiencing a large measles outbreak, in the context of multiple similar outbreaks across Europe. We devised and applied a modified chain-binomial epidemic model, requiring very simple data, to estimate the transmission parameters of this outbreak. Model results indicate sustained measles transmission among the Greek Roma population, necessitating a targeted mass vaccination campaign to halt further spread of the epidemic. Our model may be useful for other countries facing similar measles outbreaks.

Since early 2017, new measles outbreaks have been reported by several European countries [1]. While only sporadic cases were reported initially, measles has been spreading in Greece at an accelerating pace since July 2017, with a total of 1,792 cases reported between April 2017 and February 2018 (Figure 1). Of those, 896 (50%) were male and the median age was 7 years (range: 0–78). The majority of cases were reported among Greek persons of Roma descent (n = 1,136 cases; 63.7%), similar to past outbreaks [2], suggesting a significant immunity gap in this community. 

**Figure 1:**
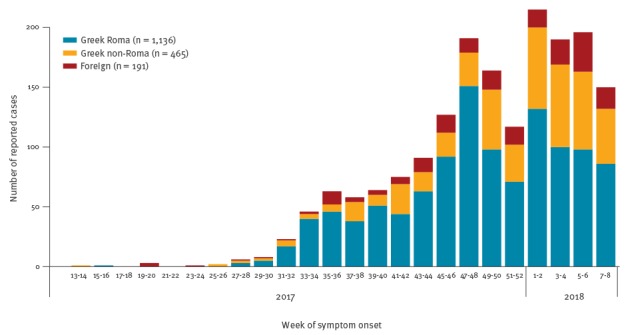
Reported measles cases by population subgroup and week of symptom onset, Greece, April 2017–February 2018 (n = 1,792)

In order to understand the transmission dynamics of the current outbreak and assess its potential impact, we implemented a modified chain binomial epidemic model fitted in a Bayesian framework. We describe here the model and its results, hoping it may be useful for other countries currently experiencing similar measles outbreaks.

## Description of the model

Chain binomial models originate from work first published in the 1950s [3] and belong to the broader class of stochastic discrete-time SIR (susceptible, infective, recovered) models. Briefly, in a population of size *N* we started from a pool of susceptible individuals *S_t_*_=0_ = *N* × *s,* where *s* is the fraction of susceptibles, with time *t* proceeding at discrete time increments equal to the generation time of the pathogen; in the case of measles, this is approximately 2 weeks [4]. The number of infective and susceptible individuals at time *t* is *I_t_* = *S_t_*_−1_ × (1 − *exp*(−*R*_0_ × *I_t_*_−1_ /*N*)) and *S_t_* = *S_t_*_−1_ − *I_t_* respectively, both being dependent solely on the number of susceptible and infective persons at the previous time step *t* − 1 [5]. *R*_0_ is the basic reproduction number, i.e. the average number of new cases produced by a single infective person in a fully susceptible population. These deterministic equations were converted to a stochastic model by considering the number of infective persons as a random variable with a binomial distribution *I_t_* ∼ *B*(*S_t_*_−1_, *p_t_*) with *p_t_* = 1 − *exp*(−*R*_0_ × *I_t_*_−1_ /*N*).

In the case of multiple population subgroups *k* = 1, 2, …, *K* of size *N_k_* each, we assumed that the intensity of transmission from cases in other groups is a fraction of that from cases in the same group. We also introduced a random effect *r* with *r* ∼ *N* (0, *σ_r_*) to model overdispersion and capture some of the heterogeneity in transmission, thereby somewhat relaxing the assumption of completely homogeneous mixing in the population [6]. Thus the infective individuals at time *t* and in group *k* became *I_t,k_* ∼ *B*(*S_t_*_−1,k_, *p_t,k_*) with $p_{t,k}=1-exp\left(-R_{0}\times exp\left(r\right)\times \left(\sum_{l=1}^{K}a_{k,l}\times I_{t-1,l}\right)/N_{k}\right)$. 

*a_k,l_* is the fraction in intensity of transmission to group *k* from cases in group *l*, with *a_k,k_* = 1 and 0 < *a_k,l_* < 1 for all k ≠ l; for simplicity we also assumed constant *a_k,l_ = A*, meaning that each case transmits to all other population subgroups with similar intensity. The exponentiated random effect exp(*r*) operates as a scaling factor, with a median value of 1, to the overall transmission intensity at every time increment and accounts for overdispersion by inflating the variance of the response variable *I_t,k_* [6]. It increases the uncertainty in the estimation of *R*_0_ and *A*, which become conditional on the random effect; its associated standard deviation *σ_r_* indicates the extent of the heterogeneity in transmission compared with the homogeneous mixing assumed in a chain binomial model.

Observed in the model were only population size and the number of infectives *I_t,k_*, which is equal to the number of reported measles cases as illustrated in the epidemic curve, with the time axis arranged in 2-week intervals (Figure 1). All other model parameters are unknown and can be estimated in a Bayesian framework, using Markov Chain Monte Carlo (MCMC), after setting appropriate prior distributions. We set *R*_0_ to a *Uniform*(10,20) prior, assuming that *R*_0_ for measles is anywhere between 10 and 20, as previously suggested [7]. For the susceptible fractions *s_k_* we used *Uniform*(0,0.4) priors, allowing up to 40% susceptibles in each population subgroup. We also set *A* and *σ_r_* to non-informative priors *A* ∼ *Beta*(1,1) and *σ_r_* ∼ *Uniform*(0.1,10). 

As set up, this model can estimate the effective reproduction number per group *R_e,k_* = *R*_0_ × *s_k_* and the number needed to additionally immunise (beyond those already immune) to achieve herd immunity *V_k_* = (1 − 1/*R*_0_) − (1 − *s_k_*), calculating these at each MCMC iteration and integrating over the chains. It can also be used to project measles cases in future weeks, as well as the final epidemic size, by sampling from multiple possible epidemic trajectories. We used the R software environment [8] to perform all calculations and JAGS (Just Another Gibbs Sampler) [9] to fit the model using MCMC. To summarise the posterior distributions for each parameter we report posterior medians and 95% credible intervals (CrI). Full JAGS and R code is available in the Supplement and at https://github.com/thlytras/measles-model, allowing our results to be replicated and the model adapted to other settings.

## Application of the model to the Greek measles epidemic

We divided the population of Greece into three groups: (i) Greek Roma, (ii) Greek non-Roma and (iii) foreign (without Greek citizenship), with estimated size *N_i_* = 300,000, *N_ii_* = 9,600,000 and *N_iii_* = 900,000 persons respectively [10,11]. To minimise reporting delay bias, we used only the measles cases reported up to week 8/2018, 3 weeks before data analysis (Figure 1); a total of 1,136 Roma, 465 non-Roma and 191 foreign cases had been reported at that time to the Hellenic Centre for Disease Control and Prevention (HCDCP) as part of disease surveillance (via the mandatory notification system). Reporting is done by physicians via a paper form (available at https://goo.gl/X9Xdwd). The model was run in JAGS using four MCMC chains with 2,000 burn-in and 30,000 sampling iterations each, without thinning.

**Figure 2:**
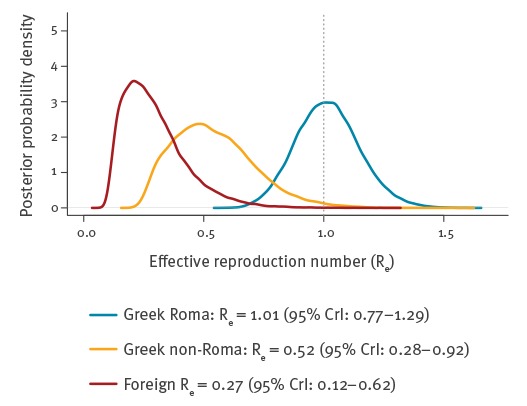
Posterior probability distributions for the effective reproduction number by population group, measles outbreak, Greece, July 2017–February 2018

The posterior distributions for the effective reproduction number are illustrated in Figure 2. Among Roma, the median *R_e_* was 1.01 (95% CrI: 0.77–1.29), while among Greek non-Roma and foreigners, it was much lower at 0.52 (95% CrI: 0.28–0.92) and 0.27 (95% CrI: 0.12–0.62), respectively. This indicates that measles is currently spreading among the Roma, whereas among the non-Roma population, there is overall adequate herd immunity. In fact, according to these results, Roma measles cases sustain and feed the epidemic in the non-Roma population, via pockets of low immunity in the latter. Estimated fractions of susceptibles in the Greek Roma, Greek non-Roma and foreign population subgroups were 7.7% (95% CrI: 4.9–11.4), 3.9% (95% CrI: 1.9–7.7) and 2.0% (95% CrI: 0.8–5.1), respectively.

CrI: credible interval; *R_e_*: effective reproduction number.

Therefore, to stop the spread of the epidemic, *R_e_* among the Roma needs to be reliably brought under 1 through mass vaccination. The percentage *V_k_* of the Roma population needed to immunise for this, as estimated by the model, is 0.1% (95% CrI: −1.9% to 2.3%); equivalently, 1.89% or 5,670 Roma should become immune to measles to bring *R_e_ < 1* with 95% certainty. These figures should be regarded only as a rough guide about the minimum scope of the overall mass vaccination effort required; in practice, several times more people will need to be vaccinated to reliably bring the epidemic under control. The main reason is that it is impossible to target only susceptible people for vaccination, and the proportion susceptible will vary significantly by age group.

**Figure 3:**
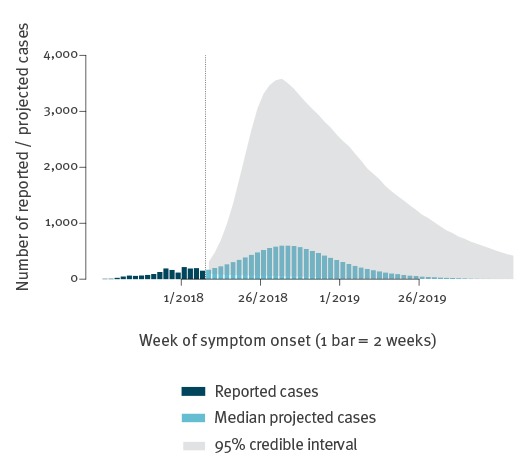
Currently reported measles cases and future model projections, all population groups, by week of symptom onset, Greece, July 2017–February 2018

The projected course of the measles epidemic according to the model, if no action were taken, is illustrated in Figure 3. It must be emphasised that imprecision is high and any epidemic trajectory within the shaded area is possible. However, in the most likely scenario, and assuming no public health action, measles cases would peak in August 2018 and then decrease until mid-2019. The estimated final epidemic size (given only as a 95% CrI) would be between 4,661 and 69,805 cases across the entire population; among Greek Roma, 2,808–23,294 cases could occur, 1,257–40,869 among Greek non-Roma and 524–8,755 among foreign persons.

**Figure 4:**
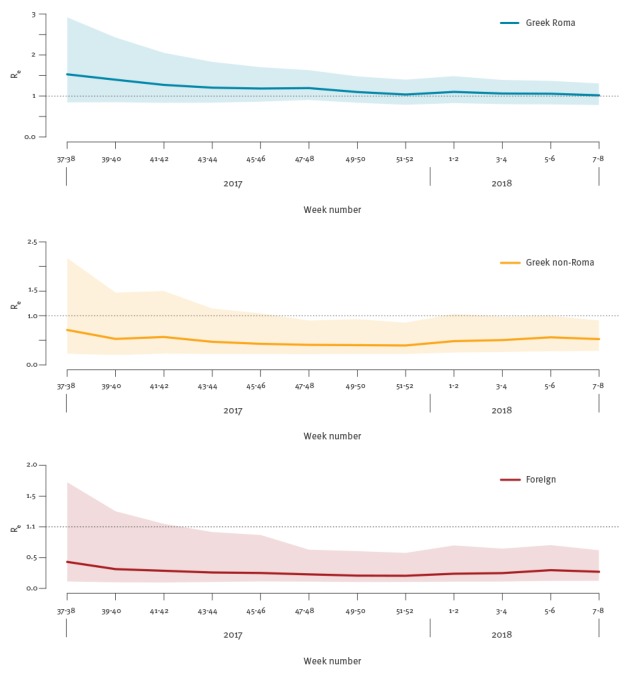
Cumulative analysis of the effective reproduction number by population group, using the data up to each consecutive bi-weekly interval, measles outbreak, Greece, weeks 37–38/2017 to 7–8/2018

As a sensitivity analysis, we applied the model repeatedly in a cumulative fashion, using the reported cases up to each bi-weekly interval from weeks 37–38/2017 to 7–8/2018. Results for *R_e_* are presented in Figure 4. The posterior medians were remarkably stable, with the 95% CrI progressively becoming narrower as more information was included in these estimates. This raised our confidence that the model, despite its simplified nature, still captures the overall dynamics of this epidemic appropriately. Full results of the model are available in the online supplement (Supplementary Table 1 and Supplementary Figures 1 and 2).

Lines: posterior medians; shaded areas: 95% credible intervals.

## Discussion

The main usefulness of this model is that it identified Greek Roma as not just the most affected group but also the driver of the current measles epidemic, sustaining it for the rest of the population as well. Recent literature has already highlighted poor vaccination uptake among Greek Roma children, less than 50% for even the first measles-mumps-rubella (MMR) vaccine dose [12]; the reasons for this are multifaceted, but it is a manifestation of the wider health and healthcare inequalities affecting this vulnerable group across Europe [13]. Our findings demonstrate that this has resulted in low population immunity among the Roma, with an average effective reproduction number near or higher than 1, allowing sustained measles transmission in this group. This initiates chains of transmission that also propagate among unvaccinated or otherwise susceptible persons in the non-Roma population, but sustained spread in that group is prevented by the existing level of population immunity (as the effective reproduction number is significantly below 1).

This finding has important practical implications for outbreak response. It indicates that public health measures targeting the general population or high-risk groups (e.g. healthcare workers), such as awareness campaigns or vaccination mandates, while useful for public health in general, will have little effect in limiting the spread of the current epidemic. An awareness campaign targeting the pool of unvaccinated persons in Greece, both Roma and non-Roma, is currently ongoing; however, based on our model, a large vaccination campaign in Greek Roma communities across the whole country is necessary to stem the current outbreak. Our model provides some informative estimates about the minimum required size of such a campaign; such efforts are currently ongoing. Furthermore, despite their low precision, future projections from the model provide insight about the epidemic to public health authorities and other stakeholders. They are very helpful to delineate the magnitude of the problem and keep minds focused on the necessity of prompt coordinated action.

Our proposed model has the important advantage that it requires only data from an epidemic curve to provide useful estimates for the transmission parameters of a measles outbreak. This simplicity is necessarily reflected in the model assumptions: neither the age structure of the population, nor geographical patterns of spread are modelled (although extensions are possible). These features of the current epidemic can be significant; most cases reported to date occurred in southern regions of Greece, and the majority of cases among Roma were children up to 14 years-old, while among non-Roma Greeks, they were adults 25 years and older (Supplementary Figures 3 and 4) [1,14]. Nevertheless, it is difficult to argue that the outbreaks in the Roma and non-Roma populations are separate and independent; this would imply a degree of social and geographic isolation that is not the case in Greece, and is also disproven by the geographic spread which is very similar among subgroups (Supplementary Figure 4). Under-reporting is similarly not modelled, but assumed constant over time and across population subgroups. Despite the oversimplification, the model appears to have described well the spread of the current measles outbreak up to this point. The accuracy of its future projections will have to be evaluated at the end of the epidemic.

In the context of the ongoing measles outbreaks affecting several European countries [1], our model is a practical tool that can easily be applied in similar settings to provide useful insight on the spread of the epidemic. It requires very few data, and can also serve as a basis for more sophisticated model extensions. Most importantly, the insight provided by applied epidemic modelling can directly inform public health action and outbreak response. As such, it deserves further attention and study.
